# Kidney function after liver transplantation: the contrasting roles of inflammation and tubular repair

**DOI:** 10.3389/frtra.2024.1480383

**Published:** 2024-10-08

**Authors:** Nina Goerlich, Seunghee Kim-Schulze, Peter Kotanko, Nadja Grobe, Xiaoling Wang, Bjoern Samans, Joe Douglas, Philipp Enghard, Paolo Molinari, Miguel Fribourg, Paolo Cravedi, Josh Levitsky

**Affiliations:** ^1^ Translational Transplant Research Center, Icahn School of Medicine at Mount Sinai, New York, NY, United States; ^2^ Department of Nephrology and Medical Intensive Care, Charité-Universitätsmedizin Berlin, Berlin, Germany; ^3^ Renal Research Institute, New York, NY, United States; ^4^ Ivana Türbachova Laboratory for Epigenetics, Epiontis, Precision for Medicine GmbH, Berlin, Germany; ^5^ Eurofins Viracor Lenexa, Lenexa, KS, United States; ^6^ Division of Gastroenterology and Hepatology, Comprehensive Transplant Center, Northwestern University Feinberg School of Medicine, Chicago, IL, United States

**Keywords:** acute kidney injury, hepatorenal, liver transplant, OPN, TIMP-1, tubular cell

## Abstract

Kidney injury is a significant complication in end-stage liver disease (ESLD), leading to increased morbidity and mortality. While liver transplant alone (LTA) can promote kidney recovery (KR), non-recovery associates with adverse outcomes, but the underlying pathophysiology is still unclear. We studied 10 LTA recipients with or without kidney failure (KF) and measured serum levels of OPN and TIMP-1 (previously identified predictors of KR), 92 proinflammatory proteins (Olink), and urinary cell populations. Our findings revealed elevated OPN and TIMP-1 levels in KF patients, strongly correlated with tubular epithelial cells in urine. Proteomic analysis showed distinct profiles in KF, non-KF, and healthy donors, indicating an ongoing proinflammatory signature in KF. Cytokines correlated with OPN and TIMP-1 levels. We propose that high pre-LTA OPN and TIMP-1 levels are crucial for tubular regeneration and normalize with kidney recovery. Insufficient pre-LTA OPN levels may lead to persistent kidney failure. Our present data also newly indicate that kidney failure post-LTA is an active condition, in which tubular cells are persistently shed in the urine. The strict association between systemic inflammation and tubular cell loss suggests a pathogenic link that could offer therapeutic opportunities to promote kidney recovery.

## Introduction

Kidney failure is a frequent complication in patients with end-stage liver disease (ESLD), significantly contributing to morbidity and mortality (1). The pathophysiology of kidney injury in ESLD is still unclear, making its management extremely challenging.

In ESLD, increased intrahepatic flow resistance due to tissue fibrosis triggers portal hypertension and vasodilation in splanchnic circulation, which reduces effective arterial blood volume (2). Reduced renal blood flow causes renal ischemia, which is thought to be one of the main mechanisms damaging tubular cells. Portal hypertension also promotes gut bacteria translocation, leading to immune system activation and systemic inflammation. On turn, systemic inflammation fuels kidney failure, through mechanisms still not fully understood.

While some patients recover kidney function after liver transplantation alone, others have persistent kidney function impairment. Understanding mechanisms that drive persistent kidney damage is crucial to developing more effective treatments and stratify patients to receive combined liver-kidney-transplantations vs. liver transplant alone (LTA).

Our prior studies showed that high circulating levels of osteopontin (OPN) and tissue inhibitor of metalloproteinases 1 (TIMP-1), are major predictors of kidney recovery after LTA (3, 4) in patients with kidney and liver failure. Of note, we also found that, in patients with kidney function recovery post-LTA, both OPN and TIMP-1 significantly declined, while they stayed elevated in patients with persistently impaired kidney function. The reasons for these different trajectories in patients with or without kidney recovery are not fully understood (4).

Herein, we hypothesize that OPN and TIMP1 may not only function as biomarker components but may, in fact, be pathogenic drivers of the regenerative vs. inflammatory/profibrotic pathways identified by the biomarker. To test this hypothesis, we used state-of-the-art technologies to measure the associations between post-LTA OPN and TIMP-1 levels in LTA recipients with or without impaired kidney function and signs of systemic inflammation and tubular cell injury.

## Methods

### Patients

For the present study, we used serum samples collected from the multicenter, prospective study, NIAID CTOT14 (National Institute of Allergy and Infectious Diseases Clinical Trials in Organ Transplantation 14; NCT01672164). The CTOT14 study prospectively enrolled 202 liver transplant (LT) recipients at seven U.S. centers to identify biomarkers of several LT complications. Informed consent was obtained from all enrolled patients under IRB approval.

Herein, we analyzed serum and urine samples collected at 1 and 2 months after transplant from 10 patients, 4 patients with post-transplant kidney failure (estimated GFR <30 ml/min) and 6 patients with normal kidney function after liver transplantation (eGFR >50 ml/min at 1 and 2 months post-transplant). GFR was estimated using CKD-EPI (Chronic Kidney Disease Epidemiology Collaboration) formula. Urine samples from 25 healthy individuals were analyzed as controls.

### OPN and TIMP-1 analyses

Serum OPN and TIMP-1 were measured by Eurofins Viracor using proprietary Luminex Bead technology and assay platform like the prior methodology published by Levitsky et al. (3) The assays performed at Eurofins Viracor uses the same antibody match pairs as the prior publication by Levitsky et al. (3) The assays were not multiplexed like the original study. OPN and TIMP-1 were measured separately in single-plex. A 5pL non-linear regression of the standard curve and sample quantification was analyzed on a Lum200 using BioPlex Manager software. The runs performed are verified with quality control samples that contain high, medium, and low concentrations of either OPN or TIMP-1 to ensure accurate sample results. Samples from the original study were used to aid in the confirmation of the method to the original study findings.

### Urine analyses

Urine (50 ml) sediment samples were processed for DNA extraction. Lysis of urinary cells was achieved by a careful combination of 67 μl lysis buffer (comprising 54.25 μl ATL buffer from Qiagen and 9 μl Proteinase K at a concentration of 30 mg/ml, CAS 39450-01-6), and 3.75 μl of a spiking plasmid essential for absolute quantification (at a concentration of 400,000 copies/μl, Genscript). This mixture underwent an incubation step at 56°C for 1.5 h, maintaining a consistent rotation speed of 900 rpm. This process ensured the accessibility of genomic DNA from urinary nucleated cells for subsequent bisulfite treatment. Bisulfite conversion was carried out by adding 270 μl of ammonium bisulfite (ranging from 65% to 75% w/w, CAS-No.: 10192-30-0) and 90 μl of tetrahydrofurfuryl alcohol (THFA, with a purity level exceeding 98%, CAS No.: 97-99-4). Following conversion, bead-based purification was performed using the Dynabeads My Silane Genomic DNA Kit from Invitrogen, ensuring the purification of bisulfite-converted DNA. To discern the specific cell types under investigation (CD3+ T cells, proximal tubular epithelial cells, and neutrophils) a qPCR-based approach utilizing demethyl-specific primers and probes tailored to cell type-specific demethylated genomic regions was employed. These primers and probes were meticulously designed to provide a high specificity for the targeted regions. Cell type-specific epigenetic markers, vital for discriminating cell populations, were identified through bisulfite-sequencing. Cell counts were calculated using the methodology established by Baron et al. (5)

### Olink analyses

Serum samples were analyzed for a panel of 92 circulating proteins associated with human inflammatory conditions using the Olink multiplex assay (Olink Target 96 Inflammation, Olink Bioscience, Uppsala, Sweden) according to the manufacturer's instructions at Icahn School of Medicine at Mount Sinai. Incubation master mix containing pairs of oligonucleotide-labeled antibodies to each protein was added to the samples and incubated at 4°C for 16 h. Each protein was targeted with two different epitope-specific antibodies to increase the specificity of the assay. Presence of the target protein in samples brings partner probes in close proximity to each other, allowing formation of a double-stranded oligonucleotide PCR target.

Data were analyzed using real-time PCR analysis software via the ΔΔCt method and NPX (Normalized Protein Expression) manager. Data were normalized using internal controls in each sample, interplate controls to normalize across plates, and a correction factor calculated by Olink from negative controls, producing NPX values proportional to the log2 of the protein concentration.

### Statistical analyses

Comparisons in urine cell populations were performed using a Mann Whitney test. For Olink cytokine results, we applied ANOVA with Benjamini & Hochberg correction for multiple comparisons. OPN and TIMP-1, and significantly different cytokines were compared between groups with *t*-test and within groups between timepoints with paired *t*-test. Pearson's correlation coefficient followed by a t distribution with length(x)-2 degrees of freedom was used to establish if the relationship between the variables was significant. All analyses were done using R version 4.3.0.

## Results

### Study population

We included 10 patients, 6 with normal kidney function (NKF) and 4 with impaired kidney function (IKF) after liver transplantation. Baseline characteristics of patients at the time of transplant were similar between the two groups, except for older age in the IKF group and higher prevalence of hypertension in the NKF group (Supplementary Table 1). Immunosuppression consisted of tacrolimus and mycophenolate. All patients in the IKF group received steroids, while only 67% of patients in the comparison group were on steroid medication. and was similar between groups. One patient in the IKF group was on mTOR inhibitor (Supplementary Table 1).

### Higher levels of OPN and TIMP-1 in IKF patients

**Figure 1:**
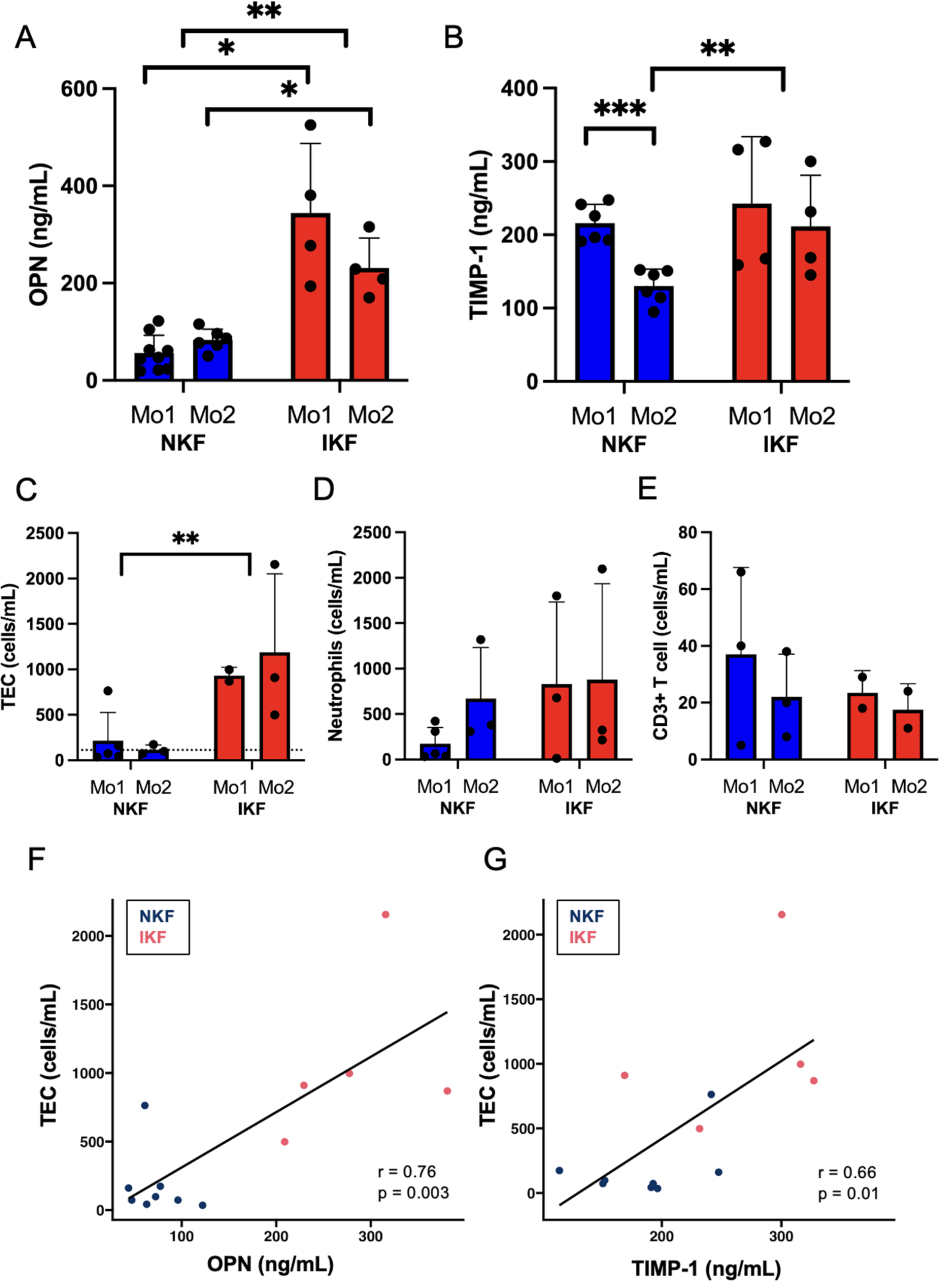
Serum levels of osteopontin (OPN) and tissue inhibitor of metalloproteinases 1 (TIMP-1) and urinary cell populations. **(A)** OPN and **(B)** TIMP-1 serum levels. **(C)** Tubular epithelial cells (TEC), dotted line represents mean of healthy controls; **(D)** neutrophils, **(E)** CD3^+^ T cells quantified in urine by epigenetic analysis. Correlation between urinary TEC and **(F)** OPN or **(G)** TIMP-1. NKF, normal kidney function after LTA; IKF, impaired kidney function after LTA; Ctrl, healthy controls *p ≤ 0.05, **p ≤ 0.01, ***p ≤ 0.001.

We first measured serum levels of OPN and TIMP-1 after LTA. In our patient cohort, IKF patients showed significantly higher OPN levels both at one and two months after LTA compared to NKF patients (Figure 1A). Patients with NKF showed a significant decrease of TIMP-1 levels between month one and month two after LTA, when they were significantly lower than in IKF patients (Figure 1B).

Overall, these data suggest that persistent kidney injury fuels production of OPN and TIMP-1, and OPN is known to be primarily produced by tubular cells in response to injury (6).

### Increased proximal tubular epithelial cells in the urine of IKF patients

Next, we quantified urinary cells by epigenetic analysis. IKF patients showed significantly increased numbers of tubular epithelial cells (TEC) in urine than patients with NKF after liver transplant at both one and two months post-liver transplant (Figure 1C). Of note, NKF patients had similar levels of TEC in the urine than healthy controls (Figure 1C, dotted line). Serum OPN levels and amounts of urinary TEC were highly correlated (Figure 1F), and serum TIMP-1 levels also correlated with urinary TEC (Figure 1G).

Urinary CD3^+^ T cells and neutrophils were not significantly different between groups, but IKF patients had numerically higher levels of neutrophils than NKF patients (Figures 1D,E).

These data indicate that patients with IKF have a persistent TEC loss in the urine, possibly due to an active inflammatory process in the kidney, while NKF is associated with full normalization of urinary cell loss.

### Proinflammatory signature in patients with IKF

**Figure 2:**
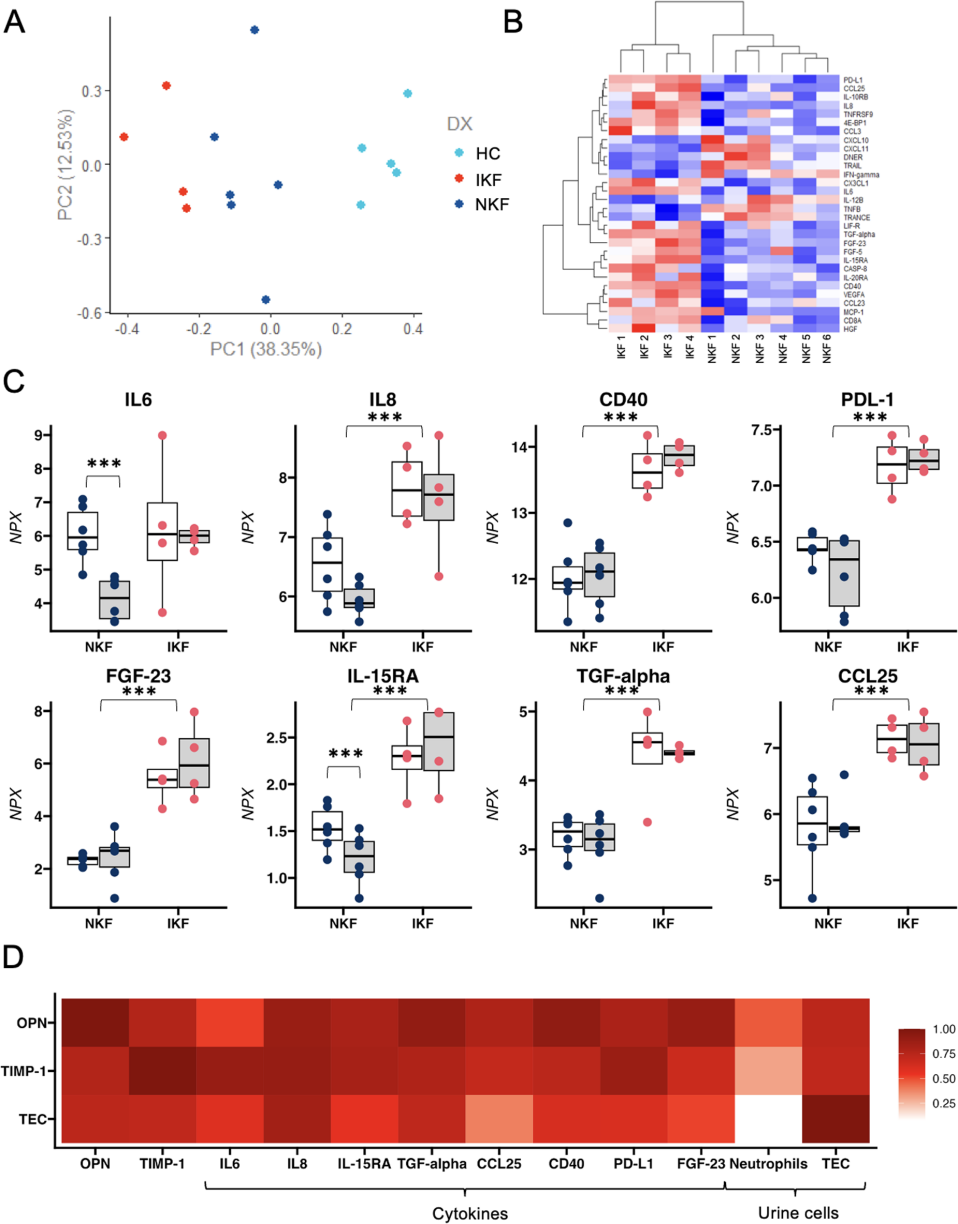
Proteomic analysis of circulating inflammatory markers. **(A)** PCA analysis reveals a distinct separation between the two cohorts of LTA patients and healthy controls based on the differential expression of 8 cytokines. **(B)** Heatmap depicting the normalized level of expression (red-blue) of representative 30 cytokines between 10 patients with LTA. **(C)** Single-patient levels of 8 cytokines that were significantly different between NKF vs. IKF patients after adjustment for multiple comparisons. **(D)** Correlation between osteopontin (OPN) and Tissue Inhibitor of metalloproteinases 1 (TIMP-1), tubular epithelial cells (TEC) and 8 significantly different cytokines and urinary cells. NKF, normal kidney function after LTA; IKF, impaired kidney function after LTA. *p ≤ 0.05, **p ≤ 0.01, ***p ≤ 0.001.

To test the hypothesis that lack of kidney function recovery is due to the counteractive effects of systemic inflammation on tubular regeneration, we performed a comprehensive proteomic analysis of circulating inflammatory markers (Olink, inflammatory panel of 92 cytokines). Principal component analysis (PCA) showed a clear distinction amongst IKF, NKF, and controls (Figure 2A). Analyses with correction for multiple comparisons showed that 8 cytokines were significantly higher in IKF vs. NKF (Figures 2B,C). Of the described changes in proinflammatory cytokines over time, IL-6 was the only cytokine that had similar levels between groups at month 1 post-transplant and then significantly declined only in patients with normal kidney function (Figure 2C). Additionally, OPN and TIMP-1 were highly correlated with the proposed cytokine signature, and urinary TEC showed strong correlations with TGF-alpha and IL-8 (Figure 2D).

In summary, our findings revealed a persistent proinflammatory profile in IKF patients that correlates with tubular cell loss in the urine, suggesting a mechanistic link between inflammation and failed kidney repair.

## Discussion

IKF patients exhibit a serum pro-inflammatory cytokine signature, which appears to abrogate the normal decrease in OPN/TIMP-1 that occurs with renal recovery. Rather, OPN/TIMP-1 remain high—as does TEC shedding—as kidney damage continues, leading to persistent kidney failure. This suggests that high levels of OPN/TIMP-1 are unable to stimulate renal recovery in a highly inflammatory environment.

OPN is a phosphoprotein that contributes to kidney tubular regeneration in different animal models of kidney ischemia (6). OPN-null mice have more pronounced ischemia-reperfusion injury (IRI) than wild-type controls, which supports a renoprotective function of OPN on TEC (7). Matrix metalloproteinases (MM) contribute to tubulogenesis during repair. TIMP-1, as a matrix metalloproteinase inhibitor, increases in response to elevated levels of MM.

In other conditions, including critically ill patients with AKI and sepsis-associated AKI, high levels of OPN and TIMP-1 herald kidney repair and decrease with successful tissue recovery (4, 8, 9). In our cohort, persistently high OPN and TIMP-1 levels, along with increased numbers of urine TEC in IKF patients, strongly support a blunted mechanism of renal recovery in the presence of pro-inflammatory plasma cytokines.

We found increased levels of IL-6, IL-8, CD40, PDL-1, FGF-23, IL-15-RA, TGF-a, and CCL25 that persist at two months after LTA in IKF patients, consistent with current concepts of inflammation as a crucial pathophysiological mechanism in the development of chronic kidney disease (10).

The strong correlations between inflammatory and regenerative markers and the amount of TEC in the urine support the hypothesis that kidney failure promotes an inflammatory milieu that, in turn, prevents tubular cell regeneration. TEC injury leads to increased OPN and TIMP-1 production and secretion, but the reparative effects of these molecules seem to underweight the damaging processes due to ongoing inflammation. In summary, our data suggest that the balance between inflammation and production of OPN and TIMP-1 as promoters of tubular repair play a critical role in determining kidney function recovery after LTA.

Despite the limited sample size, our study has noticeable strengths. All samples were collected as part of a prospective NIH-funded study (National Institute of Allergy and Infectious Diseases Clinical Trials in Organ Transplantation 14; NCT01672164), and the investigators performing the assays were blinded to patients’ outcomes. An important caveat to consider when interpreting our results is the fact that pre-transplant eGFR was already different between groups and it is possible that this contributed to the pro-inflammatory milieu that decreases the efficacy of OPN and TIMP-1 in mediating renal recovery.

## Conclusion

Persistent kidney function impairment after LTA is characterized by continuous shedding of TEC along with increased OPN and TIMP-1 levels and a proinflammatory signature. Our present data also newly indicate that kidney failure post-LTA is an active condition, in which tubular cells are persistently shed in the urine. The strict association between systemic inflammation and tubular cell loss suggests a pathogenic link that could offer therapeutic opportunities to promote kidney recovery.

## Data Availability

The original contributions presented in the study are included in the article/Supplementary Material, further inquiries can be directed to the corresponding authors.
