# Supplementary material for: Kidney function after liver transplantation: the contrasting roles of inflammation and tubular repair
Source: Front Transplant. 2024 Oct 8;3:1480383. doi: 10.3389/frtra.2024.1480383 (PMC11493771; doi:10.3389/frtra.2024.1480383)
Supplement: Supplementary file 1 [file Table1.docx]

**SUPPLEMENTARY MATERIAL**

| Variable | Total (N = 10) | NKF (N = 6) | IKF (N = 4) | p-value^1^ |
| --- | --- | --- | --- | --- |
| Age, years | 59.7 (6.8) | 56.2 (5.5) | 65 (4.7) | <0.01 |
| Female gender | 4/10 (40%) | 2/6 (33.3 %) | 2/4 (50%) | 0.24 |
| Cause of ESLD  Non-Alcoholic Fatty Liver or Cryptogenic  Hepatitis C (non-viremic)  Alcohol | 3 (30%)  5 (50%)  2 (20%) | 1 (16.7%)  3 (50%)  2 (33.3%) | 2 (50%)  2 (50%)  0 (0%) |  |
| ALT (U/L) | 25 (16.2) | 28.3 (19.0) | 22.4 (11.0) | 0.19 |
| Alkaline phosphatase (U/L) | 154.4 (126.4) | 172 (146.3) | 128 (91.7) | 0.21 |
| eGFR pre-transplant CKD-EPI  (ml/min/1.73 m²) | 63.0 (39.4) | 79.3 (30.5) | 13.9 (8.6) | <0.01 |
| eGFR post-transplant M1 CKD-EPI (ml/min/1.73 m²) | 51.9 (32.0) | 75.9 (6.2) | 15.8 (10.8) | <0.01 |
| eGFR post-transplant M2 CKD-EPI (ml/min/1.73 m²) | 47.7 (34.9) | 72.1 (19.3) | 11.0 (6.5) | <0.01 |
| Pre-transplant hypertension | 3/10 (30%) | 3/6 (50%) | 0/4 (0%) | <0.01 |
| Pre-transplant diabetes | 3/10 (30%) | 2/6 (33%) | 1/4 (25%) | 0.35 |
| Maintenance immunosuppression  Tacrolimus  MMF or MPA  Steroid  mTOR inhibitor | 10/10 (100%)  10/10 (100%)  8/10 (80%)  1/10 (10%) | 6/6 (100%)  6/6 (100%)  4/6 (67%)  0/6 (0%) | 4/4 (100%)  4/4 (100%)  4/4 (100%)  1/4 (25%) | 0.02  0.08 |

**Supplementary Table 1: Baseline characteristics of the study cohort.**

NKF, normal kidney function after liver transplantation; IKF, impaired kidney function after liver transplantation. Proportion or Mean (SD) reported as applicable; ^1^t-test.
